# Cell bank system, establishment, and application in the virus research, diagnosis, and biopharmaceutical industries

**DOI:** 10.1016/j.biotno.2025.08.001

**Published:** 2025-08-15

**Authors:** Sina Soleimani, Mohammadreza Ghorani

**Affiliations:** a Razi Vaccine & Serum Research Institute, Agricultural Research Education and Extension Organization (AREEO), P.O. Box 31975-148, Karaj, Iran; b Department of Pathobiology, Faculty of Veterinary Medicine, University of Tabriz, Tabriz, Iran

**Keywords:** Cell, Bank, System, Identification, Characterization, Certificate

## Abstract

**Background:**

The use of cells in research and diagnostic studies is important. This is of paramount importance in the biopharmaceutical product industry because cells are one of the most important platforms in the biopharmaceutical industry.

**Objective:**

The availability of highly efficient and suitable cells that can be continuously used for the mass production of biotechnological products is crucial for maintaining human health and hygiene. So, the cells used in this comprehensive system were studied, evaluated, documented, and stored for continuous and efficient use.

**Study design:**

This review article discusses all aspects of cells involved in establishing this system as a Cell Bank System (CBS). Cell banking facilities, specialized laboratories, appropriate equipment, cold rooms, cryopreservation instruments, and cell authentication and characterization are mentioned. Cell handling, row materials tests, documentation, intellectual property registration, storage, backup, and transportation were explained. In addition, policies for cell retesting and revival are discussed.

**Result:**

Based on the roadmap designed in this study, a comprehensive cell bank system can be formed for using in cell preparation, propagation, storage, and use of cells.

**Discussion:**

Considering that in the pharmaceutical industry, there is a need for a reliable, permanent, and uniform source of cells to produce and quality control the product, which can have significant effects on the products, by using this system and performing all of its aspects, diagnostic centers, researchers, and pharmaceutical manufacturers can access a reliable and persistent source of culture for related operations.

## 1 Introduction

Many pharmaceutical and biological products are produced or quality controlled through cell substrate. Therefore, cell culture plays a very important role in biotechnology industries.^1^ On the other hand, the role of cells in research and diagnostic studies is also undeniable. The most common cell lines used in biological manufacturing and research are bacterial cells, yeast cells, insect cells, plant cells, and mammalian cells.^1^ For reliable and continuous production, the cells used in the production and quality control of biological and biotechnological products must be controlled in a specialized system. This system, called the Cell Bank System (CBS), can be used to identify, document, characterize, store, and other purposes. One of the major advantages of such a system for the production of vaccines or biopharmaceuticals is the ability to have a characterized common starting source for the production Master Cell Bank (MCB) and Working Cell Bank (WCB) to produce each production lot (an MCB is defined as an aliquot of a single pool of cells that generally has been prepared from the selected cell clone under defined conditions, dispensed into multiple containers, and stored under defined conditions. The MCB is used to derive all working cell banks).^2^

The Owing to the importance of cell banking, rules and regulations for the use of cells were established many years ago.^3^ The first requirement for cell substrates was published by the WHO in 1959 for the production of an inactivated polio vaccine in primary cell cultures derived from clinically healthy monkeys.^4^ In 1960, human diploid cells (HDCs) were developed and proposed as an alternative to primary cultures of monkey kidney cells for the production of poliovirus and other viral vaccines.^4^ In 1978, the U.S. Food and Drug Administration (FDA) and Tissue Culture Association organized a meeting in Lake Placid, New York to determine whether continuous cell lines could be used in biological production.^5^ In 1986, the WHO established a study group to further investigate cell substrate issues. WHO guidelines and requirements for the use of continuous cell lines for the production of biological products were published in 1987.^6^ The concepts of MCB and WCB, and the characterization of cell substrates have been introduced in recent years.^7^

To establish a high level of confidence, many preliminary tests were performed for each vaccine series derived from human diploid cells, including testing the cell substrate for adventitious agents, karyology, and tumorigenesis.^8^

Table 1 shows the history of the evolution of cell banking technologies:

**Table 1:** Evolution of cell banking technologies: Key milestones.

Decade	General Topics	Years	Details
1950s–1960s	Foundations	1951	First immortal cell line (HeLa) established (Science).
		1955	Polio vaccine production using primary monkey kidney cells (Science).
		1960s	Advent of cryopreservation with DMSO (Technology).
1970s–1980s	Hybridomas & Early Standards	1975	Hybridoma technology (Köhler & Milstein) enables monoclonal antibody production (Science).
		1983	First Master Cell Bank (MCB) guidelines by FDA for biologics (Regulation).
		1987	WHO publishes initial cell substrate guidelines (TRS 745) (Regulation).
1990s–2000s	Recombinant Era & Automation	1990	CHO cells become gold standard for recombinant protein production (Science).
		1996	First cloned mammal (Dolly the sheep) sparks stem cell interest (Science).
		2003	Automated liquid handling robots introduced for high-throughput banking (Technology).
		2006	Induced pluripotent stem cells (iPSCs) discovered (Yamanaka, Science).
2010s	CRISPR & Global Harmonization	2012	CRISPR-Cas9 revolutionizes gene editing (Science).
		2015	FDA/EMA adopt ICH Q5D for cell substrate standardization (Regulation).
		2018	First NGS-based cell authentication guidelines (ATCC, Technology).
2020s	Personalized Medicine & AI	2020	GMP-grade iPSC banks for clinical use (e.g., CiRA, Japan) (Science).
		2022	WHO updates viral safety guidelines (TRS 1044) for cell therapies (Regulation).
		2024	AI-driven cell quality prediction tools enter use (Technology).

Many factors are involved in the establishment of a cell bank, such as the selection of appropriate tests for cell characterization, especially for the detection of cellular and adventitious contaminants in the cell bank system. In addition, banked cell substrates from mammalian cell lines used for the production of bioengineered (biotechnological-biological) products are characterized by cell identity, freedom from any cellular or microbial adventitious contaminants, presence of endogenous viral contaminants, genetic stability of the gene encoding the product, and stability over time.^9^ Therefore, this review discusses the establishment of a cell banking system. In particular, it focuses on the important aspects of cell bank establishment.

Although best practices for biobanks in general can be found in the ISBER Best Practices (now you can find the 5th edition) and ISO 20387:2018, For diagnosis purposes, there is also FDA guidance for industry, and for the biopharmaceuticals manufacturing purpose, there are also ICH guidelines, but in this article, the development of a cell bank system is explained by the precise identification of cells used in the production and quality control of biopharmaceutical products to determine their characteristics and prepare the necessary documents to control their use. Considering that each manufacturer should have its own system to form a cell bank, the purpose of this study was to examine all the factors required for the establishment of a cell bank system in biological products and manufacturing industries.^10^ Some of the information presented in this article has been obtained by the author through extensive research of published articles in this field, but most of this information is the result of the author's summaries and experiences with the cell banking system.

## 2 Objective

*The importance and application of cell banks in biotechnology:* Cell substrates have been in use in the biopharmaceutical industry for several years. One of the key principles in the production of vaccines and biopharmaceuticals that will lead to reliable and continuous production is the use of suitable cell substrates with appropriate storage. This has always been a concern for manufacturers regarding the use of cells. This issue is particularly effective in ensuring the consistency of the product.^1^ To achieve such conditions, and because of the great concerns that exist, especially among manufacturers, the cells used in the production and quality control of biological and biotechnological products must be controlled. Therefore, manufacturers that use diploid strains or continuous cell lines to produce vaccines or biopharmaceuticals typically generate, characterize, and maintain cell banks.^2^

Considering the use of cell culture in the production and quality control of many biotechnological products, as well as in virology research and diagnostic studies, it is very important to have safe and continuous cell culture. Therefore, this cell culture should be prepared, identified, characterized, documented, and stored under the right choice of tests and appropriate storage conditions.^11^

Only by developing such a cell bank, in which all the cases mentioned in this article are taken into account, is it possible for the biotechnological products and production industry to produce with specific quality based on international requirements, planned quantity, and demands to achieve continuous production. Therefore, it is very important to consider these issues when developing a cell bank and using it in the industry.^12^

## 3 Study design

**Fig. 1:**
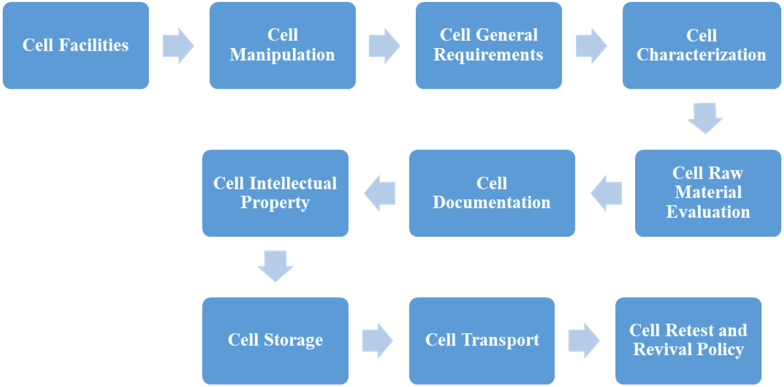
Cell bank establishment diagram.

To establish a cell banking system in a center, the following 10 principles must be studied, designed, and implemented step-by-step (Fig. 1):

### 3.1 Cell facilities

**Fig. 2:**
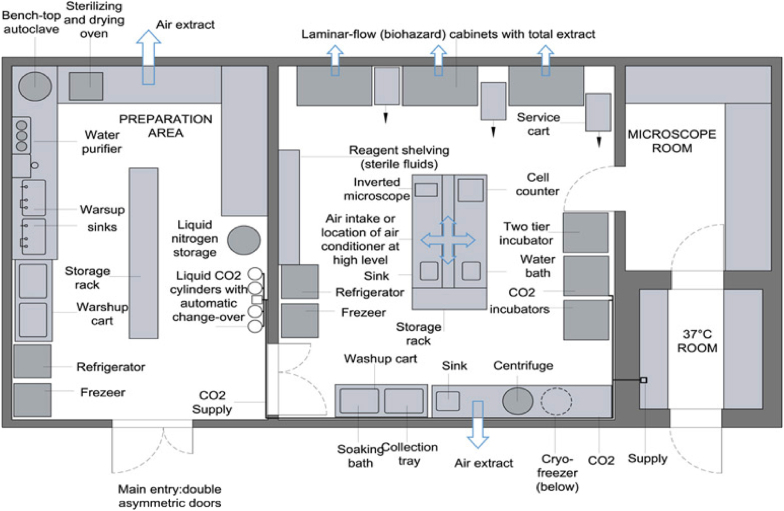
Schematic illustration of a typical cell culture laboratory with the location of the required equipment.

The first step in designing, building, and establishing a cell banking system is to have good facilities. These facilities include a dedicated cell laboratory that meets the relevant requirements (Fig. 2) and biosafety situations, and a +4 °C cold room for setting up nitrogen tanks.^13^

Additionally, appropriate cell culture equipment should be used. All the equipment needed to handle the cell from beginning to end, as well as the nitrogen tanks needed to store the cell, must be prepared and used. Nitrogen tanks should be stored in a cold room at +4 °C.^13^ In general, a modern cell bank consists of the following parts:

1. **Cell Processing Area (Class II Biosafety Cabinet Zone)**

1-1 **Biosafety Cabinets (BSCs):**

o **Type A2:** For non-hazardous cell lines (e.g., CHO, HEK293).
o **Type B2:** For viral vector work or primary human cells (100 % exhaust).
o **Placement:** Away from doors/air vents to minimize airflow disruption.

1-2 **Support Equipment:**

o CO_2_ incubators (dual-stack for redundancy).
o Benchtop centrifuges (with aerosol containment lids).

2. **Quality Control Lab**

2-1 **Testing Stations:**

2-2 **Microscopy:** Phase-contrast for viability checks.
2-3 **PCR Hood:** Dedicated space for mycoplasma testing.
2-4 **Flow Cytometry:** Cell phenotype/identity confirmation.

3. **Cryopreservation & Storage Zone**

3-1 **Liquid Nitrogen (LN**_**2**_**) Storage Configuration:**

o **Vapor-Phase LN**_**2**_ **Tanks:** For master/working cell banks (segregated racks for MCB vs. WCB).
o **Backup LN**_**2**_ **Supply:** Alarm-monitored with auto-refill (per FDA 21 CFR Part 11).
o **Redundancy:** Dual storage in separate fireproof rooms (EMA compliance).

3-2 **Controlled-Rate Freezers:** Adjacent to BSCs for direct vial transfer.

4. **Regulatory & Documentation Wing**

4-1 **Electronic Batch Records (EBR):** Secure servers for chain-of-custody logs.
4-2 **Sample Archive:** −80 °C freezers for retained QC samples.

5. **Support Infrastructure**

5-1 **Air Handling:** HEPA-filtered, positive pressure in clean zones, negative in BSL-2 areas.
5-2 **Emergency Power:** Backup generators for LN_2_ tanks/freezers.

### 3.2 Cell manipulation

The cells required should be obtained from reliable national and international sources. Therefore, these sources should be properly investigated and identified. Cell banks are classified into two types based on their purpose and role in biotechnological research and manufacturing. The two primary types of cell banks in biotechnology manufacturing are the master cell bank and the working cell bank, each of which serves different functions in the preservation and utilization of cell lines. End-of-production cell lines are generated at the end of production and are important for quality control and reference. The master cell bank is a critical starting point of the cell banking system. It contains a large number of well-characterized and validated cells that serve as primary sources for the generation of working cell banks and subsequent production cell lines. The master cell bank is a fundamental element in biotechnological development, which ensures that the same cell line is used consistently throughout the life cycle of a product. The working cell bank is derived from the master cell bank, and serves as an immediate source for production purposes. It provides sufficient quantities of cells for research, development, and manufacturing processes.^14^

To use the cell and develop a cell bank, after the cell enters the laboratory, necessary preparations must be made to prepare a certain number of master and working cells according to the laboratory schedule. In addition, cell handling should be defined. The most important issue in cell culture laboratories is to follow the necessary rules for cell handling so that the cells studied are not contaminated, destroyed, or in the unsuitable conditions. Therefore, the principles of cell manipulation should be considered.^15^^,^^16^

#### 3.2.1 Preparation of master and working cells

Cells obtained from international or national centers were used for cell preparation. The following scheme should be considered when preparing a suitable reserve for a cell:

#### 3.2.2 Required number of cells

The number of tubes should be planned according to the needs of the laboratory.

#### 3.2.3 Prevention of cross-contamination

Cell cross-contamination is one of the most important considerations that must be taken into account when working with cells and during the storage period. To this end, the operator should take the maximum precautions necessary when working with cells. Operations were performed based on specific requirements under controlled conditions and in an appropriate laboratory environment. Therefore, laboratories should have a specific policy on this issue entitled the “Cell Contamination Prevention Policy”.^17^^,^^18^

#### 3.2.4 Avoid reduction of cell viability

During cell manipulation, conditions must be created that have the least impact on cell viability. Therefore, conditions such as temperature, materials and environments used, and type and amount of serum required must be carefully controlled and planned.

#### 3.2.5 Appropriate cell filling

For the preparation of required cells, the type and shape of the cryotubes used, as well as the method of filling the cryotubes, must be considered, which should be done in the best possible way using durable cryotubes.^19^

#### 3.2.6 Clone selection

Initial screening is crucial in the development of cell lines, as it helps in selecting clones with the best growth, productivity and stability. This process identifies clones with ideal characteristics for the production of high-quality biological products. For monoclonal antibodies and recombinant proteins, selection of high-yielding and stable clones is essential to meet product requirements. In gene and cell therapies, clone selection is used to accurately identify genetic alterations and functional properties of cell lines. Advanced techniques, such as single-cell cloning and high-throughput screening, are used to select clones that not only meet regulatory requirements but also ensure the efficacy and safety of the final product.

By integrating robust clone selection strategies, biopharmaceutical companies can increase the reliability and consistency of their manufacturing processes and deliver safer and more effective treatments to patients. Furthermore, establishing cell banks based on selected clones ensures that high-quality characteristics are maintained.^20^

CRISPR/Cas9 technology enables the rapid generation of loss-of-function mutations in a targeted gene in mammalian cells. A single cell harboring those mutations can be used to establish a new cell line, thereby creating a CRISPR-induced knockout clone. These clonal cell lines serve as crucial tools for exploring protein function, analyzing the consequences of gene loss, and investigating the specificity of biological reagents.^21^

### 3.3 Cell general requirements

One of the most important aspects of developing a cell banking system is the creation of a certificate for the desired cell, which consists of two main parts. The first is the collection and processing of basic and general information regarding the cell. It should be noted that this information plays an important role in cell research and evaluation. The second part is the cell characterization, which will be discussed in later sections.

Therefore, all general and basic information about the cells should be collected and analyzed. This information will help vaccine manufacturers and researchers to understand the target cell and how to use it. The general cell requirements include the following groups of identity information^10^:

#### 3.3.1 General cell information

General information of the cell as the first step in the initial identification of the cell includes the following information that must be collected, determined, and documented for each cell:

The type of cell, including primary cell types, diploid cells, cell lines, and stem cells, is one of the basic types of cells studied. In addition, the shape of the cell during growth, which is one of the three states of sticky, semi-sticky, or floating, should be specified. In this section, the tissue source of the desired cell and the host species of the tissue are specified, that is, from which tissue and from which organism the cell was obtained. Other information in this section includes the name of the cell supplier, cell's primary code, primary identification number of the cell from the supplier, and media used during the cell growth, storage, and maintenance phases.

#### 3.3.2 Cell specifications

One of the most important cell specifications is the accurate data on cell passage history. The first was to determine the desired cell passage stage. From this perspective, the cells are divided into two stages: The Master and the Working cells. Some laboratories have added another stage, called the parent cell, to the cell passage stage before the master cell. In addition, the passage information of the cells, including the passage number and date, must be determined. Considering that cell passage information is very important for cell efficiency, the information in this step will be very effective for using the cell, especially in the production process.^22^

#### 3.3.3 Cell storage conditions

This section records all the cell storage information for all three cell phases. This information includes the storage date, number of nitrogen tanks, and canister number. This information should be provided separately for the primary and backup storage tanks of a cell. The type of preservative used for cell storage and the operator's name were also recorded at this stage.^23^

#### 3.3.4 Quantitative cell information

Further information to be provided is quantitative information about the cells. Therefore, the number of cell cryotubes of cells stored and the number of cells in each cryotube, obtained by counting cells and determining cell viability, as explained in the following sections, should be determined.

#### 3.3.5 Coding and labeling

Coding and labeling at each stage of cell passage must be performed under the internal rules and regulations of the laboratory or manufacturer. The cell identification code must include the following minimum information, such that the selected code has a specific identity:

Cell name or abbreviation of the cell name, cell passage stage by mentioning all three stages or abbreviation of the names, cell passage number, source of the cell preparation, year of preparation of the desired cell, batch number of the cell preparation, and the number of cell cryotubes compared to the total number of tubes prepared in that batch. The cell label should convey information about the cell so that an independent identity can be created for the cell label with defined colors. The designed code is engraved on the label together with other specifications to form the cell label.

### 3.4 Cell characterization

**Fig. 3:**
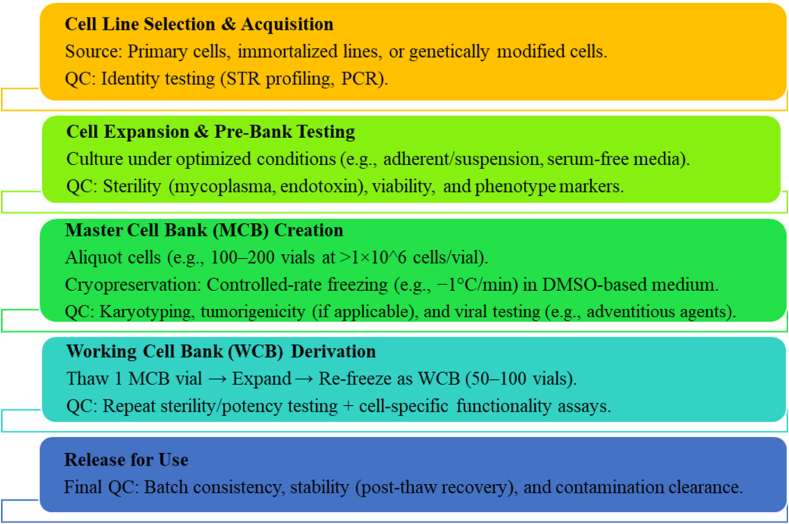
Establishment of a master/working cell bank (MWCB).

For the second part of the cell certificate, the necessary tests to determine the cell characteristics should be performed, and the results recorded. The test results can be used to make decisions and plan how, when, and for how long the study can be used. Determining the characteristics of the cells in the cell bank at the time of initial entry into the bank or at any other planned time will help the manufacturer plan the use of the desired cell. The most important discussion in this section is the selection of the correct test and the proper and valid performance of the desired test. The most important tests required to determine cell characteristics are as follows^24^^,^^25^ (Fig. 3):

#### 3.4.1 Cell identification

**Fig. 4:**
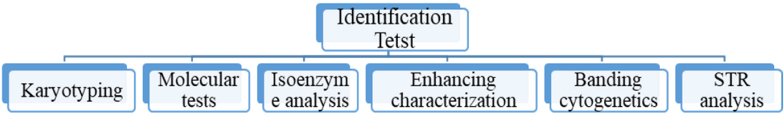
Cell identification tests.

6 basic tests should be performed in this group (Fig. 4):

##### 3.4.1.1 Karyotyping

A chromosomal map of the cell can be constructed by performing cell karyology. The origin of the desired cell can be confirmed based on a chromosomal map of the cell.^26^

##### 3.4.1.2 Molecular tests

By designing primers for the origin of the desired cell, the identity of the cell can be investigated and evaluated using molecular methods.^27^ Molecular techniques are used to analyze the genetic material of cultured cells, providing insights into gene expression, mutations, and viral infections. Molecular tests used to identify cell cultures include:

PCR and qPCR: These techniques amplify specific DNA or RNA sequences, enabling detection and quantification of genetic material, such as viral genomes or gene expression levels.

DNA Sequencing: Provides detailed information about the genetic code of cells, allowing for the identification of mutations, variations, and genetic abnormalities.

Microscopy and Image Processing: Fluorescence microscopy and image analysis can be used to visualize cellular structures, monitor cell behavior, and assess the effects of drugs or other treatments.

##### 3.4.1.3 Isoenzyme analysis

Isoenzyme analysis by electrophoresis is the recommended technique for cell line authentication. Several studies have introduced molecular techniques for cell line authentication and have even suggested replacing isoenzyme techniques. The genetic polymorphisms of enzymes observed by electrophoretic mobility can distinguish cell lines from different animal species. Isoenzyme analysis for cell line authentication began with the vertical starch gel technique. Subsequently, most studies used agarose electrophoresis film from a commercially available kit.^28^

##### 3.4.1.4 Cell enhancing characterization

Today, in addition to traditional methods of cell identification, advanced methods are also used for this purpose. One of these methods is next-generation sequencing. Next-generation sequencing (NGS) is an advanced technology that enables comprehensive genetic analysis of cell lines. Unlike traditional cell identification methods that rely on in vivo and in vitro assays and are often time-consuming and have limited sensitivity, NGS provides rapid and accurate characterization of cell lines while meeting stringent regulatory requirements. However, to fully exploit the potential of NGS in cell line characterization, it is essential to integrate genetic analysis and robust data management.

While traditional methods such as PCR and Sanger sequencing have been widely used to identify cell lines, they often require multiple assays to achieve comprehensive results. However, NGS offers a more accurate and comprehensive approach that replaces or complements traditional methods. NGS can provide whole genome sequencing for identification, metagenomic sequencing for purity, and copy number variation analysis for stability. NGS technologies are versatile and can be used for a variety of purposes and replace traditional methods.^29^

##### 3.4.1.5 Banding cytogenetics

For human or animal cells grown in a confluent manner, morphological analysis can be a useful tool alongside other tests. In most cases, isoenzyme analysis is sufficient to confirm the species of origin for cell lines derived from human or animal sources. Depending on the history of the cell line, other tests may be appropriate. Other advanced technologies may be used to confirm the species of origin, such as cytogenetic banding or the use of species-specific antisera. This method uses DNA analysis to detect a pattern of genomic polymorphisms (e.g., restriction fragment length polymorphisms, variable number of tandem repeats, or genomic dinucleotide repeats) to detect a unique marker chromosome. Confirmation of the species of origin or the presence of known unique cell line markers is considered an appropriate test for identification. Expression of the product of interest may be a complementary approach to confirm identity.

##### 3.4.1.6 STR (Short tandem repeat) analysis

STR is another advanced technique to identify and authenticate cell lines by analyzing specific, highly variable regions of DNA. It's a standard technique for ensuring cell line identity, verifying purity, and detecting cross-contamination in cell cultures. STRs are short, repeating sequences of DNA (typically 2–7 base pairs long) that vary in length between individuals and cell lines. STR analysis involves amplifying specific STR loci (locations on the DNA) using PCR (Polymerase Chain Reaction) and then analyzing the amplified fragments, often using capillary electrophoresis. This method used for o confirm the identity of a cell line by comparing its STR profile to a reference database or known profile, identify the presence of contaminating cells from other cell lines and ensure the stability and purity of cell lines used in research.^30^ In Table 2, Cell authentication techniques were compared:

**Table 2:** Comparison of cell authentication techniques.

Parameter	Traditional: STR Profiling	Advanced: NGS (Whole Genome/Exome)
**Principle**	Amplification of short tandem repeat (STR) loci (e.g., 8–16 loci).	Sequencing of entire genome/exome or SNP panels.
**Sensitivity**	Moderate (can miss low-level contamination <10–20 %).	High (detects contamination <5 %, SNPs, CNVs).
**Resolution**	Limited to predefined STR loci.	Genome-wide (detects mutations, drift, cross-species contamination).
**Throughput**	Low to moderate (1–2 days for 10–20 samples).	Moderate to high (3–7 days, but scalable for 100s of samples).
**Cost per Sample**	$50–$150 (low reagent cost).	$200–$1000 (depends on depth/coverage).
**Standardization**	Well-established (ATCC, DSMZ, CLSI guidelines).	Evolving (lack of universal thresholds for NGS).
**Data Complexity**	Simple (electropherogram peaks).	Complex (requires bioinformatics pipelines).
**Best Use Case**	Routine QC for cell banks (MCB/WCB release testing).	Research/diagnostics

#### 3.4.2 Cell growth profile

**Fig. 5:**
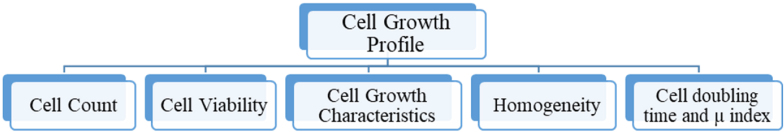
Cell growth profile.

Assays to assess cell growth can be performed as follows^31^^,^^32^ (Fig. 5):

##### 3.4.2.1 Cell total count

This test determines the number of cells in the cryotube. Based on the cell counts, the next plan for cell use and cell passage can be determined.

##### 3.4.2.2 Cell viability test

This test determined the percentage of viable cells. Cells viability can be assessed using trypan blue exclusion, and unstained cells were counted.

##### 3.4.2.3 Cell growth characteristics

The desired cell growth characteristics can be evaluated by counting cells and generating cell growth curves at different hours of logarithmic growth. The microscopic appearance of the cell substrate should be stable within the master cell bank.

##### 3.4.2.4 Homogeneity

In this type of study, the mode of growth, uniformity of growth, and method of cell growth in the desired system should be examined and evaluated.

##### 3.4.2.5 Cell doubling time and μ index calculation

By counting cells at specific time intervals, cell doubling time and the μ index can be calculated.

#### 3.4.3 Cell tumorigenic properties

Two main tests are performed in this group of tests. In these tests, the possible tumorigenic properties of the cells under study should be determined.

##### 3.4.3.1 Tumorigenicity

The purpose of this test is to determine whether the cells of interest are capable of producing tumors when inoculated into laboratory animals. This test is necessary to ensure that the continuous cell lines used in the manufacturing of biological products are not tumorigenic. A description of the tumorigenic properties of cells is required for all diploid and non-diploid cells, but is not required for primary cell cultures that are not subcultured or that are subsequently subcultured for only a very limited number of population doublings.^33^

##### 3.4.3.2 Oncogenicity

In addition to Tumorigenicity testing, Oncogenicity testing should be performed on cells used to manufacture biological products. Oncogenicity testing is required whenever test cells are tumorigenic in an immunosuppressed animal model.^34^, ^35^, ^36^

#### 3.4.4 Cell stability studies

Stability studies play an important role in the evaluation of cells, especially since they ensure the efficacy of the cells as well as the identity of the cells during storage, especially long-term storage, as well as during various processes of cell use. The studies divided into two groups.

##### 3.4.4.1 Genetic stability study

The main concerns regarding the cell substrate stability are the consistent production of the recombinant (rDNA) protein and the maintenance of the production capacity during storage under defined conditions. For this purpose, an analysis of the genetic stability of the production cells (MCB) and cells at the limit of in vitro cell age (End of Production Cells) should be compared.^7^ In Table 3 the differences between regulatory organizations are summarized in the topic of genetic sustainability:

**Table 3:** Regulatory requirements for cell banking: Genetic stability testing.

Agency	Guideline	Genetic Stability Threshold	Required Tests	Key Differences
**FDA**	CFR 21, ICH Q5D	**MCB:** ≤10 % karyotypic abnormality.	Karyotyping (passage-matched to production).	Focus on product consistency; NGS accepted but not mandated.
		**WCB:** No additional drift beyond MCB.	STR profiling (for identity).	
**EMA**	EMA/CHMP/BWP/532517/2008	**MCB/WCB:** "No clinically relevant alterations."	Karyotyping + SNP arrays (if engineered).	Stricter on therapeutic relevance of mutations.
		Post-production: ≤15 % aberrant metaphases.	Tumorigenicity testing (if applicable).	
**WHO**	TRS 962 (Annex 3)	**MCB:** ≤5 % polyploidy/aneuploidy.	Karyotyping + FISH (for viral integration).	Emphasizes viral safety alongside stability.
		**WCB:** Comparable to MCB.	Isoenzyme analysis.	
**ICH**	ICH Q5D (Global Harmonization)	**MCB:** "No adverse impact on safety/efficacy."	Karyotyping (≥20 metaphases analyzed).	Least prescriptive; defers to regional authorities.
**PMDA**	JP XVII (Japan)	**MCB/WCB:** ≤10 % abnormality (strict for iPSCs).	NGS encouraged for pluripotent cells.	Most aggressive in adopting NGS

##### 3.4.4.2 Storage stability studies

In these studies, the level of cell stability was determined, especially in terms of viability during the storage period of the cell, particularly during long-term storage. Cells should be sampled at specific time intervals, and cell viability can be calculated. It is best to extract a cryotube of cells from the nitrogen tank every three months for at least one year and determine the cell growth profile by performing the following tests:

Cell total count, cell viability, cell growth characteristics and cell homogeneity. If there is a significant change in any of the above tests during any period of test time, the cell does not have the required stability.

#### 3.4.5 Determination of cell purity

One of the most important aspects of cell health is the contamination of cells by various unwanted factors that contaminate cells, which plays a crucial role in the efficiency and productivity of cells in both industry and laboratories. The purity of the cell under study and any possible unwanted contaminations can be determined through the following processes^12^:

##### 3.4.5.1 Evaluation of bacteria and fungi in cells

Cells can be evaluated for bacterial and fungi contamination by culturing the them in microbiological media.

##### 3.4.5.2 Evaluation of adventitious viruses in cells

This can be done through in vivo and in vitro tests.

###### 3.4.5.2.1 In vivo tests

In vivo tests involve culturing cells in laboratory animals, such as adult mice, suckling mice, guinea pigs, rabbits, and embryonated chicken eggs.

###### 3.4.5.2.2 In vitro tests

In vitro tests are performed by culturing specific cell types. Additionally, special serological or molecular tests and transmission electron microscopy (TEM) may be employed to detect particular viruses in the cells.

In some cases, researchers may also utilize antibody production tests to identify viruses. The most significant bovine viruses recommended for detection in cells include bovine viral diarrhea virus (BVD), bluetongue virus, bovine respiratory syncytial virus (BRSV), bovine adenovirus, bovine parvovirus, reovirus 3, and rabies virus. Retroviruses, which are particularly significant, can be tested using infectivity assays, reverse transcriptase (RT) assays, and transmission electron microscopy studies.^37^

Human cell substrates are screened for human viral pathogens such as Epstein–Barr virus, cytomegalovirus, human retroviruses (human T-lymphotropic virus [HTLV] and human immunodeficiency virus), hepatitis B and C viruses, human herpesviruses (HHV-6 and -7), parvovirus B-19, polyomaviruses (JC virus [JCV] and BK virus [BKV]), and human papillomaviruses (HPV-16 and -18). These screenings can be conducted through appropriate in vitro techniques or PCR analysis.^24^

##### 3.4.5.3 Assessment of mycoplasmas and mycobacteria

The likelihood of cell contamination by mycoplasmas and mycobacteria is assessed by culturing the cells in specific media. Molecular tests, like qPCR, are used also to detect and eliminate mycoplasma contamination.

By completing the above evaluations, a comprehensive certificate of the examined cells is developed, which can be stored in the Cell Bank System.^10^

### 3.5 Cell raw material evaluation

In cell culture, laboratories utilize various raw materials for cell growth, storage, and maintenance. Therefore, to establish an effective cell banking system, manufacturers and researchers should test and certify these materials. In particular, animal-derived raw materials used as supplements in the cultivation of cell substrates must be tested for bacteria, fungi, mycoplasma, and viruses. Regarding transmissible spongiform encephalopathy, certification should be provided to confirm that the animal-derived reagents are free from this agent.^7^ The most important raw materials used in cell culture include the following:

#### 3.5.1 Serum

Sera are among the most critical raw materials used at various stages of cell culture and are integral to the process. Key types of sera include calf serum (CS), fetal bovine serum (FBS), human serum albumin (HSA), bovine serum albumin (BSA), and fetal calf serum (FCS). The following tests should be performed to evaluate serum quality: sterility, mycoplasma, mycobacteria, and bacterial endotoxin tests to detect microbial contamination. Additionally, tests for hemoglobin, passage doubling time (PDT), cytotoxicity, and performance via plating assays are conducted to assess serum quality. Other physicochemical tests include pH, appearance, total protein, and osmolality. For adventitious virus detection, specific tests should screen for bovine viral diarrhea virus (BVDV), parainfluenza-3 (PI3), bovine herpesvirus-1 (BHV-1), and infectious bovine rhinotracheitis (IBR).^38^

#### 3.5.2 Trypsin

The species from which trypsin is derived (used in vaccine production, including cell banks and viral seed production) should be clearly identified. Both bovine and porcine-derived trypsin must be tested in accordance with established recommendations.

#### 3.5.3 Amino acid

The source of amino acids used in the growth medium should be documented.

#### 3.5.4 Other biological reagents

Other biological reagents used during cell culture, such as transferrin, insulin, antibiotics, DMSO, and other growth factors in growth media, should be tested for their species of origin. These reagents must be obtained from reliable sources and accompanied by valid certificates from the supplier company. Importantly, penicillin should not be added to the viral vaccine production substrate, and other beta-lactam antibiotics must be excluded from production cell cultures.

### 3.6 Cell documentation

The establishment of a cell bank system requires the preparation, compilation, archiving, and management of all necessary documents for cell control. Documentation is a crucial component of a reliable cell bank. The required documents for cell control in the cell bank system must include specifications, instructions, guidelines, protocols, forms, test records, and other essential documentation^39^:

#### 3.6.1 Cell certificate

A key requirement for establishing a cell bank is the preparation of a cell certificate, which is based on cell information and the results of various characterization tests. The cell certificate consists of two main parts: general information about the cell, derived from the identification of its general characteristics, and cell characterization, which is based on the results of characterization tests. An example of the cell certificate in two phases are presented in Table 4, Table 5.

**Table 4:**
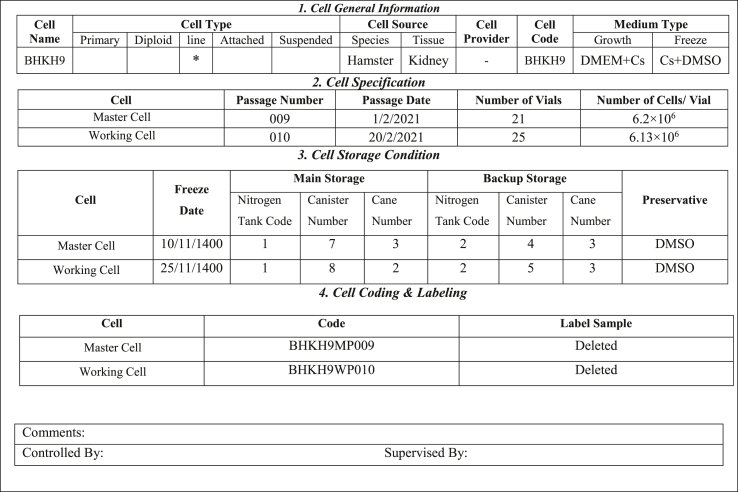
A example of cell certificate (Phase 1) (Cell General Requirements) 1. Cell General Information.

**Table 5:**
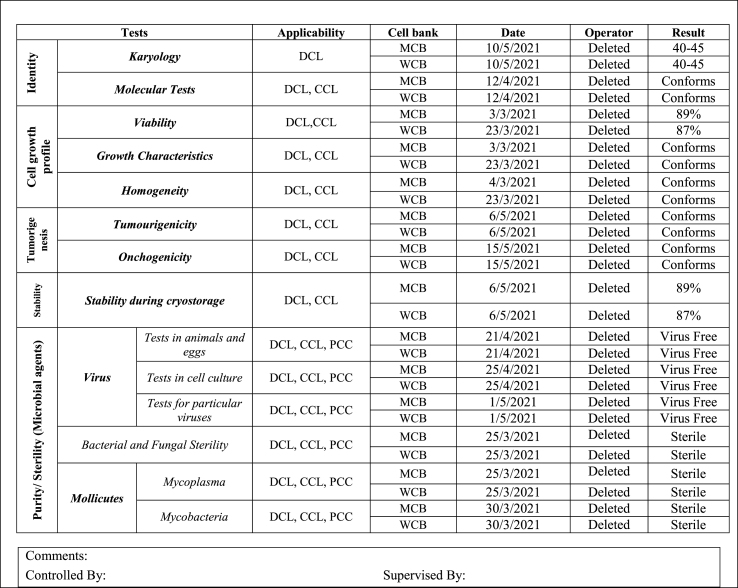
A example of cell certificate (Phase 2) (Cell Characterization).

#### 3.6.2 Instructions and protocols

All necessary instructions, protocols, and guidelines for cell manipulation, maintenance, storage, and transportation must be developed according to regulatory and internal requirements.^40^

#### 3.6.3 Test records

The necessary forms to record the information obtained from the tests to determine the characteristics of the cells under study should be designed in this system, and all the test results should be recorded on the forms.

#### 3.6.4 Specification

It is essential to extract and document all specifications for the tests necessary to determine the characteristics of the cells and raw materials, applying them to various testing processes.

### 3.7 Cell intellectual property

After you obtained cell lines from a cell bank, you are not allowed distribute them to any third party. Distribution of cell lines to third parties is strictly prohibited in the material transfer agreement concluded at the time of cell distribution.

For a cell to be recognized as belonging to a system, particularly in the case of newly discovered cells, it must be classified within the intellectual property system as the property of the relevant organization or individual.^41^^,^^42^ This consideration is especially important for new cells developed by researchers or custodian centers, ensuring that the intellectual property rights to the cell are assigned to the respective researcher or center that introduced it. Of course, this does not apply to cells that have already been used and are in public use.

To facilitate this process, it is necessary to submit the newly introduced cell along with the relevant documentation to the intellectual property system. After going through the legal procedures and review of the claimed invention and associated documents, the outcome will be communicated. If the invention is approved, it will be registered, and a patent certificate will be issued in the name of the researcher or the center responsible for the invention. This intellectual property system may be national or international.

### 3.8 Cell storage

Considering that cell storage is one of the most critical factors affecting cell quality and efficiency, all storage requirements must be clearly defined and adhered to. During the storage period, cell storage conditions must be continuously monitored, and any potential issues must be addressed immediately. Proper storage significantly impacts cell viability, so all requirements based on the specific conditions of the cells should be carefully considered. Three main issues should be addressed when storing cells^43^:

#### 3.8.1 Cell freezing (cryopreservation)

Cryopreservation required to preserve cell viability and functionality at extremely low temperatures, typically −80 °C to −196 °C. There are some advanced cell freezing techniques, includes:

- Vitrification, is a rapid cooling technique that avoids ice crystal formation by transforming the cell solution into a glassy, non-crystalline state.
- Droplet Vitrification, a variation of vitrification, droplet vitrification involves freezing cells in small droplets to enhance cooling rates.
- Ice Crystal Inhibition Techniques, researchers are exploring the use of some methods to inhibit ice crystal formation during cooling.
- Programmable Cryopreservation, controlled-rate freezers are used to gradually cool cells at a specific rate, typically around −1 °C per minute, until they reach −70 °C to −80 °C.^21^^,^^44^

You must choose the appropriate method based on the conditions of the desired cell. Selection of cryopreservation methods based on cell type and scale is done as follows:

1. Cell Type

**1-1- Sensitive cells (e.g., iPSCs, primary cells):** Vitrification (ultra-rapid cooling) to avoid ice crystallization.
**1-2- Robust cells (e.g., CHO, HEK293):** Controlled-rate freezing (standardized, scalable).

2. Scale of Banking

**2-1- Small-scale (research):** Vitrification (manual, high viability for limited vials).
**2-2- Large-scale (GMP):** Controlled-rate freezing (automated, batch consistency).

3. Downstream Use

**3-1- Diagnostics (high viability needed):** Optimize recovery (e.g., post-thaw supplements).
**3-2- Bioproduction (scale-up):** Prioritize cost-effectiveness and reproducibility.

Table 6 shows the cell viability with different cryopreservation protocols:

**Table 6:** Recovery rate efficiency.

Cell Type	Slow Freezing	Vitrification	LN_2_ Direct
**HEK293**	95 %	98 %	40 %
**CHO**	85 %	75 %	35 %
**iPSCs**	60 %	90 %	20 %

#### 3.8.2 Cold room

The cold room for nitrogen tank storage should maintain a temperature of +4–8 °C. This temperature must be controlled and monitored using a data logger. If the temperature rises, technical personnel should investigate the issue immediately, resolve any problems, and continue monitoring the cold room until it returns to the desired temperature. If the issue persists and the temperature exceeds +15 °C, the nitrogen tanks containing the cells should be transferred to a designated backup cold room that has reached the appropriate temperature.

#### 3.8.3 Nitrogen tanks

Nitrogen tanks are used for the cryopreservation of cells at extremely low temperatures, typically below −150 °C (often around −180 °C). The nitrogen tanks for basic cell storage should be standard, and their volume should be planned based on the number of cell tubes. One of the most critical factors in storing cells in nitrogen tanks is the nitrogen level, which must be checked regularly. Nitrogen is supplied in two ways: continuous and non-continuous. In a continuous system, nitrogen is automatically injected into the tanks, and the operator must monitor the nitrogen level. In a non-continuous system, the nitrogen level is measured periodically (at most once a week) by an operator using specialized rulers, based on factors such as tank capacity, opening diameter, and the number of cells. If the liquid nitrogen level falls below the specified range, additional nitrogen must be added to the tanks.^45^

#### 3.8.4 Documentation of cell storage

All storage procedures and any issues encountered during the storage period must be documented. These records should be maintained in the cell laboratory archive for a specified duration.

#### 3.8.5 Cell backup

To prevent the loss of important cells, the cell bank system must have provisions for retaining a percentage of cells as backups. This ensures that, in the event of an incident, the stored backup can be utilized. Manufacturers should implement two backup systems: one within another tank (to safeguard against accidents with the main tank) and another in a different location (to protect against incidents in the building housing the nitrogen tanks). The first is referred to as Internal Backup, while the second is called External Backup. Both types of backups should be considered, especially for sensitive and critical cells, and all storage requirements for the main cells should also apply to backup cells.

### 3.9 Cell transport

Since transporting cells from international centers to laboratories or between research centers and institutions that manufacture biological products are often necessary, the method of cell transport significantly impacts the quality and efficiency of the cells. Therefore, all requirements for cell transport must be compiled and adhered to during the transfer process.^46^ ISO 21973 also provides relevant points for cell transportation. When transporting cells, the following considerations should be taken into account:

#### 3.9.1 Cell transport documents

All documents used to identify and characterize the cell, particularly the cell certificate, along with any precautionary documents, must be prepared and sent with the cells. Additionally, the Cell Transfer Form, as shown in Table 7, should be completed.

**Table 7:**
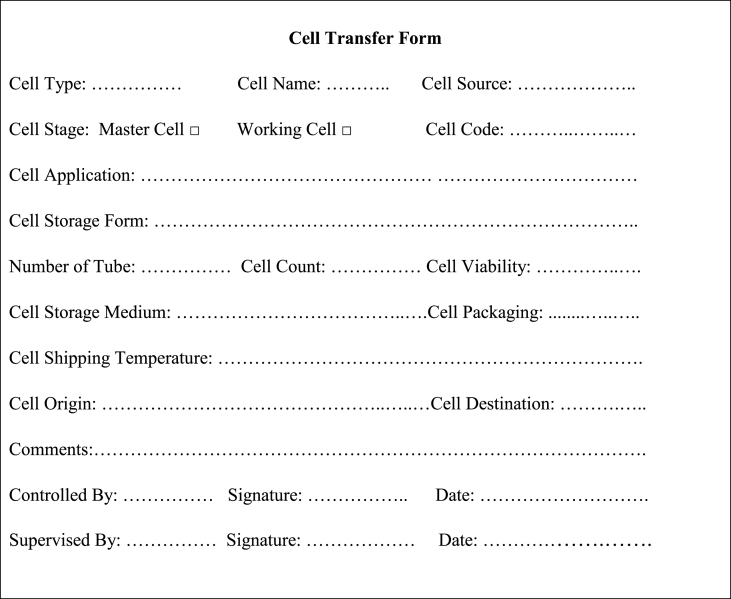
The cell transfer form.

#### 3.9.2 Cell transport packaging

**Fig. 6:**
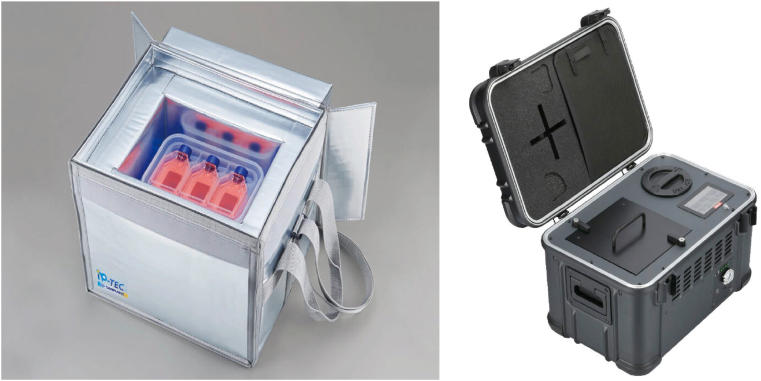
Live cell Shipper (for cell flask and cell cryo tube).

Proper packaging is crucial for cell transfer to preserve the cells and protect them from environmental conditions. The packaging must be tailored to the storage form of the cells, ensuring appropriate conditions for transport. An example of suitable packaging for biological samples is illustrated in Fig. 6.

#### 3.9.3 Cell transport temperature and time

Cells should be transported in the shortest possible time while maintaining the cold chain. Portable nitrogen tanks are preferred for transporting cells. If cells are transferred in flasks, they should be moved quickly and kept within a temperature range of 25–30 °C. Frozen cells must be transported in portable nitrogen tanks to ensure their stability.

### 3.10 Cell retest policy and revival

One of the most critical aspects of a cell bank, particularly for its longevity, is the establishment of a well-formulated policy for periodic and case-specific testing, along with a plan for reviving weakened cells. Laboratories should conduct basic tests to determine the characteristics of cells at planned intervals. Additionally, any damaged cells should be tested and resuscitated, if necessary, in the event of issues with cell storage and maintenance.

Cells should undergo routine testing for viability, identity confirmation, and genetic and storage stability. Quality control tests must be performed to verify the absence of adventitious agents, ensuring that the stored cells remain free from harmful pathogens.

After long-term storage, cells may experience reduced viability. Therefore, based on the results of cell characterization tests, it is essential to revive the cells by culturing them in an appropriate medium using specific procedures at designated time intervals after confirming a loss of viability.

## 4 Conclusion

A cell banking system is a systematic process for preserving and storing cell lines under controlled conditions for future use in various biotechnological applications, such as virological research, diagnostics, and the manufacture of biological products.^13^ The primary goal of cell banking is to maintain a renewable and stable supply of well-characterized cells, ensuring their viability and functionality over extended periods.

Some of the advantages of using a cell bank system are as follows:

• *Reproducibility and Continuity:* Ensures consistent availability of cell lines for research and production.
• *Accelerated Development:* Facilitates faster development and production of drugs and biological products, aiding clinical trials.
• *Cost Savings:* Reduces costs associated with repetitive cell line production.
• *Standardization and Scalability:* Promotes standardized research practices and scalability in production.^47^

A well-structured cell banking system guarantees an adequate supply of equivalent, well-characterized cells throughout the expected lifecycle of biological products. Besides providing consistent biological starting material, it allows for thorough characterization of the cell substrate, minimizing risks of adventitious agent contamination and maximizing the chance of detecting potential contaminants. Establishing a well-characterized cell banking system is essential for the quality control program in the manufacturing process of biological products. This ensures compliance with Good Manufacturing Practices (GMP).^48^^,^^2^

The first step in designing a cell banking system involves evaluating and identifying the target cell. The characteristics and growth conditions of the cell substrates significantly influence the properties and safety of the final biological products. Therefore, a comprehensive understanding of these characteristics is vital to identify critical issues and to develop a quality control system that addresses them.^7^^,^^49^

Describing a cell's characteristics is essential not only for assessing its capabilities but also for verifying its authenticity. Depending on the intended purpose, cell investigation and identification can be performed using various techniques.^50^

Documentation of cells is another foundational aspect of developing a cell bank. To utilize a specific cell in the biopharmaceutical industry, comprehensive cell documents must be prepared based on the results of characterization tests.

When developing a cell bank, additional factors must be considered, including:

Suitability of the laboratory, Equipment's for cell culture, Reliable sources for cell procurement, Compliance with culturing requirements, Intellectual property registration, Appropriate storage facilities and Transportation procedures.

Each of these elements should be carefully designed, planned, and implemented according to established standards. Effective planning and execution can lead to a well-organized system for the preparation, preservation, storage, and use of cells in research studies, diagnostics, and the production of biological products.

This review article outlines the fundamental principles of cell bank development, providing a roadmap for manufacturers and researchers to establish an effective cell banking system. Based on the roadmap designed in this study, a comprehensive, documented and continuous cell bank system can be formed for using in cell preparation, propagation, storage, and use of cells and biopharmaceutical manufacturers can maintain a reliable and efficient cell bank consistently.

Due to the importance of using cell culture for the production of biological products as well as basic research and diagnosis of many diseases, the use of cell culture is expanding day by day, so in the future, the establishment of a cell bank system in every production and research center is inevitable so that an efficient system is constantly available. Also, cell culture techniques, especially passage techniques and identification and characterization of cell cultures, are expanding so that in the future, we will witness the design of faster and more accurate techniques for determining the characteristics of cell cultures that will replace existing methods.

## CRediT authorship contribution statement

**Sina Soleimani:** Writing – original draft, Supervision, Project administration, Methodology, Investigation, Funding acquisition, Formal analysis, Data curation. **Mohammadreza Ghorani:** Writing – review & editing, Writing – original draft, Supervision.

## Declaration of competing interest

All authors agree to declare that they have no conflicts of interest.

## References

[1] Tekarslan Şahin Ş., Mesut B., Ozsoy Y. (2017). Applications of cell culture studies in pharmaceutical technology. Acta Pharm Sci.

[2] Sobolewska-Ruta A., Zaleski P. (2019). Cell banks preparation in biopharmaceuticals production. Adv Microbiol.

[3] Hilleman M.R. (1968). Cells, vaccines, and the pursuit of precedent. Natl Cancer Inst Monogr.

[4] WHO. Requirements for Poliomyelitis Vaccine (Inactivated) (1959).

[5] Petricciani J.C. (1991). Regulatory philosophy and acceptability of cells for the production of biologicals. Dev Biol Stand.

[6] WHO (1987). WHO Expert Committee on Biological Standardization. World Health Organization, Annex 3.

[7] Hayflick L. (2001). A brief history of cell substrates used for the preparation of human biologicals. Dev Biol.

[8] Perkins F.T. (1971). Suggested methods for the management and testing of a passaged diploid cell culture used for virus vaccine production. Jpn J Med Sci Biol.

[9] Tirino V., Paino F., d’Aquino R., Desiderio V., De Rosa A., Papaccio G. (2011). Methods for the identification, characterization and banking of human DPSCs: current strategies and perspectives. Stem Cell Rev Rep.

[10] Soleimani S. (2024). Cell identification, characterization, and documentation for use in the production of biological products. Arch Razi Inst.

[11] Perpiñá U., Herranz C., Martín-Ibáñez R. (2020). Cell Banking of HEK293T cell line for clinical-grade lentiviral particles manufacturing. Transl Med Commun.

[12] Feng F., Huang C., Xiao M. (2020). Establishment and characterization of patient-derived primary cell lines as preclinical models for gallbladder carcinoma. Transl Cancer Res..

[13] Wrigley J.D., McCall E.J., Bannaghan C.L. (2014). Cell banking for pharmaceutical research. Drug Discov Today.

[14] Froud S.J., Jenkins N. (1999). Animal Cell Biotechnology: Methods and Protocols.

[15] Ziaiifar F., Soleimani S., Lotfi M. (2020). Characterization of the BHK-21C5 cell line and its introduction for use in research, diagnostics and production of biological products. Alborz Univ Med J.

[16] Ziayaeifar F., Soleimani S. (2022). Evaluation of BHK-21 cell line for specific viruses by two different PCR methods. Arch Razi Inst.

[17] Adombi C.M., Lelenta M., Lamien C.E. (2011). Monkey CV1 cell line expressing the sheep-goat SLAM protein: a highly sensitive cell line for the isolation of peste des petits ruminants virus from pathological specimens. J Virol Methods.

[18] Capes-Davis A., Theodosopoulos G., Atkin I. (2010). Check Your Cultures! A List of Cross-Contaminated or Misidentified Cell Lines. Int J Cancer.

[19] Thomas S., Huynh-Ba K., Huynh-Ba K. (2010). Pharmaceutical Stability Testing to Support Global Markets.

[20] Aeschlimann S.H., Graf C., Mayilo D. (2019). Enhanced CHO clone screening: application of targeted locus amplification and next-generation sequencing technologies for cell line development. Biotechnol J.

[21] (2024). Hong T., Bae S.M., Song G., Lim W. Guide for generating single-cell–derived knockout clones in mammalian cell lines using the CRISPR/Cas9 system. Mol Cells.

[22] Fus-Kujawa A., Prus P., Bajdak-Rusinek K. (2021).

[23] Buick E., Mead A., Alhubaysh A. (2024). CellShip: An Ambient Temperature Transport and Short-Term Storage Medium for Mammalian Cell Cultures. Biopreserv Biobank.

[24] Schiff L.J. (2005). Review: production, characterization, and testing of banked mammalian cell substrates used to produce biological products. In Vitro Cell Dev Biol Anim.

[25] Weiskirchen S., Schröder S.K., Buhl E.M., Weiskirchen R.A. (2023). Beginner’s Guide to Cell Culture: Practical Advice for Preventing Needless Problems. Cells.

[26] Uno Y., Nozu R., Kiyatake I. (2020). Cell culture-based karyotyping of orectolobiform sharks for chromosome-scale genome analysis. Commun Biol.

[27] Ziyaeifar F., Soleimani S., Lotfi M. (2021). Identification of Iranian BHK-21-C5 cell line by two steps polymerase chain reaction. Arch Razi Inst.

[28] AraÚJo S.B.D., Patricio G.F., Simoni I.C., Rivas E.B., Fernandes M.J.B. (2019). Isoenzyme and molecular approach for authenticating and monitoring of animal cell lines. An Acad Bras Cienc.

[29] Chen Y.-H., Connelly J.P., Florian C., Cui X., Pruett-Miller S.M. (2023). Short tandem repeat profiling via next-generation sequencing for cell line authentication. Dis Model Mech.

[30] Almeida J.L., Korch C.T. (2023). Assay Guidance Manual.

[31] Patil R., Kale A.D., Mane D.R., Patil D. (2020). Isolation, culture and characterization of primary cell lines of human buccal mucosal fibroblasts: a combination of explant enzamytic technique. J Oral Maxillofac Pathol.

[32] Yashwanth B.S., Goswami M., Kooloth Valappil R., Thakuria D., Chaudhari A. (2020). Characterization of a new cell line from ornamental fish *Amphiprion ocellaris* (Cuvier, 1830) and its susceptibility to nervous necrosis virus. Sci Rep.

[33] Sato Y., Bando H., Di Piazza M. (2019). Tumorigenicity assessment of cell therapy products: the need for global consensus and points to consider. Cytotherapy.

[34] Furesz J., Fanok A., Contreras G., Becker B. (1989). Tumorigenicity testing of various cell substrates for production of biologicals. Dev Biol Stand.

[35] Oh S., Gu E.Y., Han J.S. (2022). Tumorigenicity assessment of human cancer cell lines xenografted on immunodeficient mice as positive controls of tumorigenicity testing. Int J Toxicol.

[36] Gutiérrez-Martínez A., Sew W.Q.G., Molano-Fernández M., Carretero-Junquera M., Herranz H. (2020). Mechanisms of oncogenic cell competition-Paths of victory. Semin Cancer Biol.

[37] Torabi S., Soleimani S., Mahravani H., Ebrahimi M.M., Shahsavandi S. (2023). Mouse fibroblast L929 cell line as a useful tool for replication and adaptation of infectious bursal disease virus. Arch Razi Inst.

[38] Shi L., Zhang X., Yu Y. (2018). Quality control of bovine serum in viral vaccines. J Appl Virol.

[39] Soleimani S. (2024). Cell identification, characterization, and documentation for use in the production of biological products. Arch Razi Inst.

[40] Soleimani S., Abedi Kiasari B. (2012). Evaluation and comparison of Hela, Hep2C and Vero cell lines sensitivity to polio vaccinal virus using micro and macro vaccine potency tests. Arch Razi Inst.

[41] Cyranoski D., Contreras J.L., Carrington V.T. (2023). Intellectual property and assisted reproductive technology. Nat Biotechnol.

[42] Saha C.N., Bhattacharya S. (2011). Intellectual property rights: an overview and implications in pharmaceutical industry. J Adv Pharm Technol Res.

[43] Mirabet V., Alvarez M., Solves P., Ocete D., Gimeno C. (2012). Use of liquid nitrogen during storage in a cell and tissue bank: contamination risk and effect on the detectability of potential viral contaminants. Cryobiology.

[44] Jaiswal A.N., Vagga A., Jaiswal A., Vagga A.A. (2022). Cryopreservation: a review article. J Chemometr.

[45] Bajerski F., Nagel M., Overmann J. (2021). Microbial occurrence in liquid nitrogen storage tanks: a challenge for cryobanking?. Appl Microbiol Biotechnol.

[46] Miller P.G., Wang Y.I., Swan G., Shuler M.L. (2017). A simple cell transport device keeps culture alive and functional during shipping. Biotechnol Prog.

[47] Heidemann R., Lünse S., Tran D., Zhang C. (2010). Characterization of cell-banking parameters for the cryopreservation of mammalian cell lines in 100-mL cryobags. Biotechnol Prog.

[48] Soleimani S. (2022). A review of the establishment of the seed lot system in the production of biological products and its importance. Arch Razi Inst.

[49] WHO (2013). Replacement of Annex 1.

[50] del Puerto-Sardiñas C.A.H.-F.M., González-Rodríguez H., Leyva-Rodríguez A.L. (2016). Seed Lot System from Neisseria meningitidis strains cultured in non-animal origin media. VacciMonitor.

